# Prognostic analysis of uveal melanoma based on the characteristic genes of M2-type macrophages in the tumor microenvironment

**DOI:** 10.1186/s12859-023-05396-9

**Published:** 2023-07-11

**Authors:** Li Fu, Qun Huang, Yongfeng Wu, Diang Chen

**Affiliations:** 1 Department of Ophthalmology, Jian Yang Hospital of Traditional Chinese Medicine, Chengdu, Sichuan China; 2 Department of Ophthalmology, Hospital of Chengdu University of Traditional Chinese Medicine, Chengdu, Sichuan China; 3 Department of Andrology, Hospital of Chengdu University of Traditional Chinese Medicine, Sichuan Chengdu, China

**Keywords:** Uveal melanoma, M2 macrophages, Tumor microenvironment (TME), The Cancer Genome Atlas (TCGA), Gene expression omnibus (GEO)

## Abstract

Uveal melanoma arises from stromal melanocytes and is the most prevalent primary intraocular tumor in adults. It poses a significant diagnostic and therapeutic challenge due to its high malignancy and early onset of metastases. In recent years, there has been a growing interest in the role of diverse immune cells in tumor cell development and metastasis. Using The Cancer Genome Atlas and the gene expression omnibus databases, and the CIBERSORT method, we investigated the topography of intra-tumor immune infiltration in uveal melanoma in this research. We evaluated the prognosis of uveal melanoma patients using the M2 macrophage immune cell infiltration score in conjunction with clinical tumor patient data. We built a prognostic model based on the distinctive genes of M2 macrophages and combined it with patients’ clinical data in the database; we ran a survival prognostic analysis to authenticate the model’s accuracy. The functional study revealed the importance of macrophage-associated genes in the development of uveal melanoma. Moreover, the reliability of our prediction model was verified by combining tumor mutational load, immune checkpoint, and drug sensitivity, respectively. Our study provides a reference for the follow-up study of uveal melanoma.

**Supplementary Information:**

The online version contains supplementary material available at 10.1186/s12859-023-05396-9.

## Introduction

Uveal melanoma develops in the stroma’s melanocytes and is the most prevalent primary intraocular tumor in adults [1]. Up to 50% of patients with primary uveal melanoma eventually develop distant metastases. The preferred location for this extremely malignant tumor is the posterior pole of the eye. It is vulnerable to metastasis by the transmural stream, with a dismal prognosis for 85% of cases metastasizing to the liver. The median survival is reported to be 4–5 months [2]. Uveal melanoma can originate from melanocytes anywhere in the uveal tract. About 85–90% arise from the choroid, with the remainder confined to the iris or ciliary body [3]. Most uveal melanoma has metastasized by the time of detection, and the treatment of metastatic uveal melanoma is now limited by the absence of a viable systemic medication. The tumor microenvironment (TME) is crucial in the development, progression, metastasis, and recurrence of melanoma. Immune, inflammatory, endothelial, and mesenchymal cells are among the several non-tumor and stromal cell components present in uveal melanoma TME [4]. Previous studies have shown that pro-angiogenic tumor-associated macrophages (TAM) promote homing, extravasation, and metastasis to the liver in uveal melanoma [5].

Macrophages are involved in numerous homeostatic and disease processes in the body. With effector activities including phagocytosis, antigen presentation, and flexibility in the secretion of various signaling molecules, they serve as an efficient “firewall” in controlling homeostasis in the body [6]. In recent years, it has been found that there are two different cell polarization patterns of macrophages, the classical polarization pathway and the alternative polarization pathway, resulting in pro-inflammatory M1-type macrophages and anti-inflammatory, pro-proliferative, and pro-tumor M2-type macrophages [7]. The diverse immune cells’ identification in carcinogenesis and the investigation of diagnostics and therapy processes have received increasing attention due to tumor immunity research [8, 9].

The extensive use of second-generation sequencing technologies has increased the emphasis on genetic and molecular explanations and studies of tumor cell development [10–13]. This study explored the intra-tumor immune infiltration landscape in uveal melanoma using The Cancer Genome Atlas (TCGA) and Gene Expression Omnibus (GEO) databases and the CIBERSORT algorithm. We evaluated the prognosis of uveal melanoma patients based on the M2 macrophage immune cell infiltration (ICI) score and clinical data from tumor patients, constructed a prognostic model by characterizing genes in M2 macrophages, and validated the accuracy of our predictive model by combining tumor mutational load, immune checkpoints, and drug sensitivity, respectively. To provide a reference for the follow-up study of uveal melanoma.

## Methods

### Data retrieval and collation

Eighty uveal melanoma patients’ RNA sequence data and clinical characteristics were gathered from The Cancer Genome Atlas (TCGA) database, and these samples served as a training set. For validation, an independent cohort GSE22138 was selected from the Gene Expression Omnibus database (GEO, https://www.ncbi.nlm.nih.gov/geo/), which contains data from 63 uveal melanoma cases. The processing comprised downloading the raw data, annotating the probe, complementing missing values, and eliminating inter-P discrepancies. Two expert bioinformatics analysts handled the processing of this data.

### Immune cell infiltration analysis

We applied the CiberSort algorithm to analyze the immune cell infiltration in 22 samples from each training set [14], Each tumor sample’s relative immune cell infiltration content was determined, and M2 macrophage infiltration data were collected to serve as the basis for the following study. Co-expression analysis was used to acquire the clinical data and M2 macrophage-related gene expression in uveal melanoma samples in the TCGA database, establishing the groundwork for the subsequent analysis.

### Functional enrichment analysis

To identify the pathways of M2 macrophage-related genes, we performed functional enrichment analysis of GO, KEGG, and GSEA for M2 macrophage-related genes. Additionally, we used protein interaction network analysis to clarify the potential correlations between M2 macrophage-related genes and uveal melanoma to investigate the relationships between these genes.

### Build prognosis model

Single-factor Cox regression analysis was conducted in the training team to screen potential prognostic genes. In Cox regression analysis, significant (p < 0.05) genes were considered potential prognostic genes. The median risk score served as the dividing line between the low- and high-risk patient groups in the training queue.

### Survival analysis

Survival differences between high and low-risk groups were analyzed by ROC analysis to assess the prognostic ability of genetic traits further. Combining the samples and doing independent survival analyses for different sexes and stages, we examined the model’s performance across various subgroups to further validate its predictive accuracy.

### Survival analysis of clinical subgroups

By integrating the sub-permits of the clinical data, we grouped the clinical data of the sample. Further, we evaluated the predicted outcomes of the prognostic model amongst the various subgroups.

### Progression free survival (PFS) analysis

Combining the pan-cancer clinical data from the TCGA database with the risk values of the samples we generated from our model, we further analyzed the progression-free survival disparities between high- and low-risk groups by dividing the median risk values into high- and low-risk groups.

### GSEA functional enrichment analysis

GSEA functional enrichment analysis was performed by combining the risk value of each sample and the gene expression matrix in the samples. After filtering parameters were established, the more obvious pathways in the high and low-expression groups were chosen as the next phase in the study based on the pathways with differential expression between the high and low-risk groups in the results.

### Immune checkpoint correlation analysis

We performed co-expression analysis by the immune checkpoint-related gene expression and the samples’ risk values to obtain immune checkpoint genes correlated with the samples’ risk.

### Analysis of differences in tumor mutational load

Using the tumor mutation load data of the samples in the TCGA database, combined with the risk values of the samples, we analyzed the differences in mutation load in high and low-risk groups, analyzed the mutation differences of modeled genes between high and low-risk groups, and further revealed the tumor mutation mechanism.

### Drug sensitivity analysis

We assessed each sample for drug sensitivity in conjunction with the database’s data files on drug sensitivity, and we then included the sample’s risk values to compare the sensitivity of high- and low-risk groups to various drugs.

## Results

**Fig. 1:**
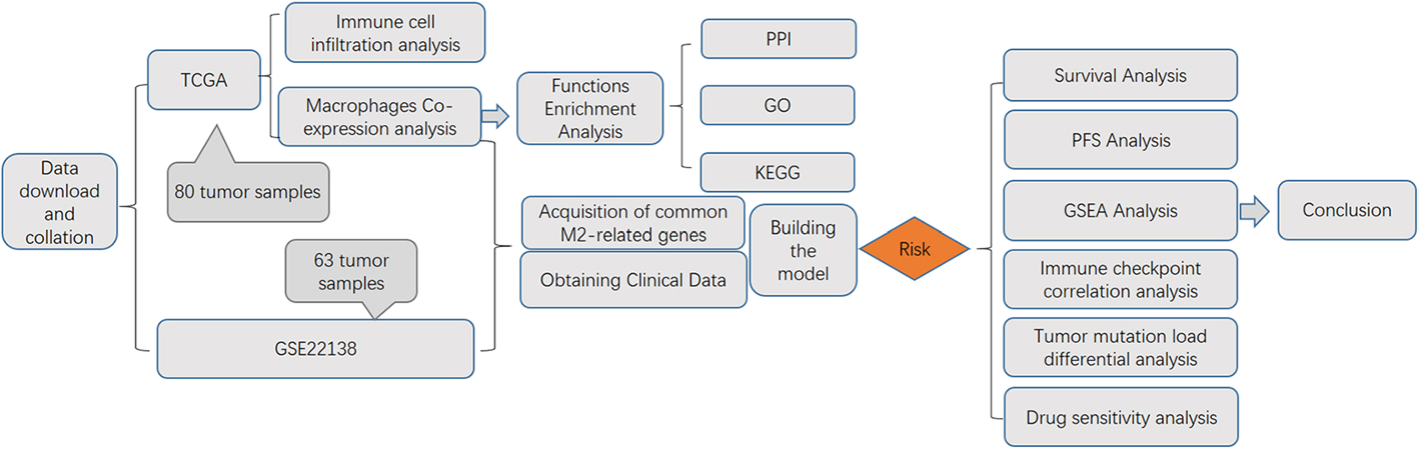
Flow chart of the entire study

The detailed flow chart of our study is shown in Fig. 1.

### Co-expression analysis results

**Fig. 2:**
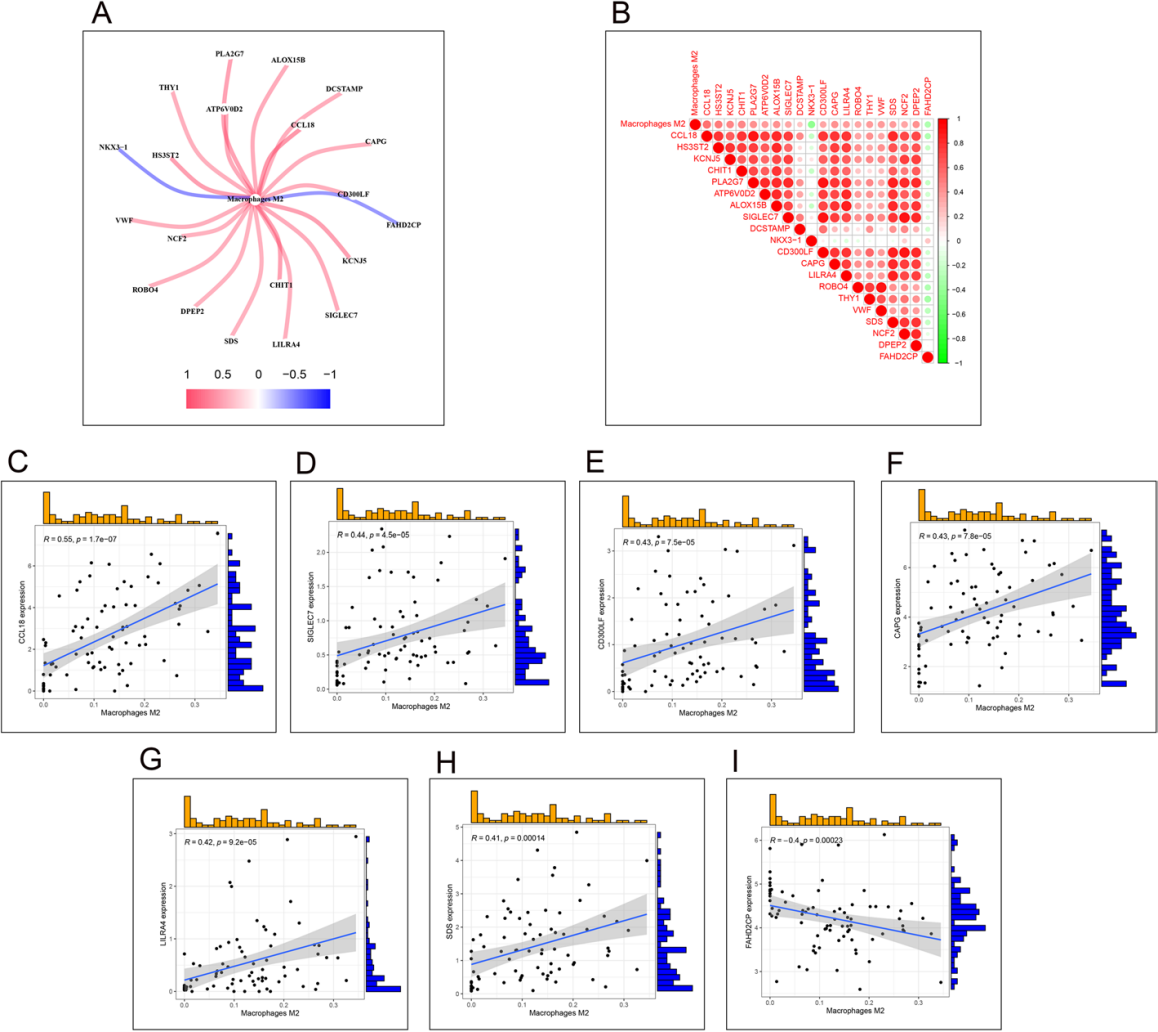
Co-expression analysis results. ** A**, **B** Results of co-expression of M2 macrophages -related genes with M2 macrophages ,18 genes positively associated with M2 macrophages and 2 two genes negatively associated with M2 macrophages. **C**–**I** represents the results of co-expression of 7 target genes (CCL18, SIGLEC7, CD300LF, CAPG, LILRA4, SDS, and FAHD2CP) with M2 macrophages

We performed immune cell infiltration analysis in 22 samples of each training set by the CiberSort algorithm, combined with the characteristic genes of M2 macrophages, filtered by correlation coefficient and p-value (filtering condition of correlation coefficient of corFilter = 0.4, pFilter = 0.05 filtering condition for correlation test p-value). Briefly, 20 M2 macrophage-related genes were obtained, and these genes’ expression in the samples was extracted for the subsequent analysis. The results are displayed in Fig. 2A, B. According to the co-expression network diagram, 18 genes are positively associated with M2 macrophages, and two are negatively associated with them. We separately extracted the seven target genes (CCL18, SIGLEC7, CD300LF, CAPG, LILRA4, SDS, and FAHD2CP) for the modeling application. The results are shown in Fig. 2C–I.

### Functional enrichment analysis

**Fig. 3:**
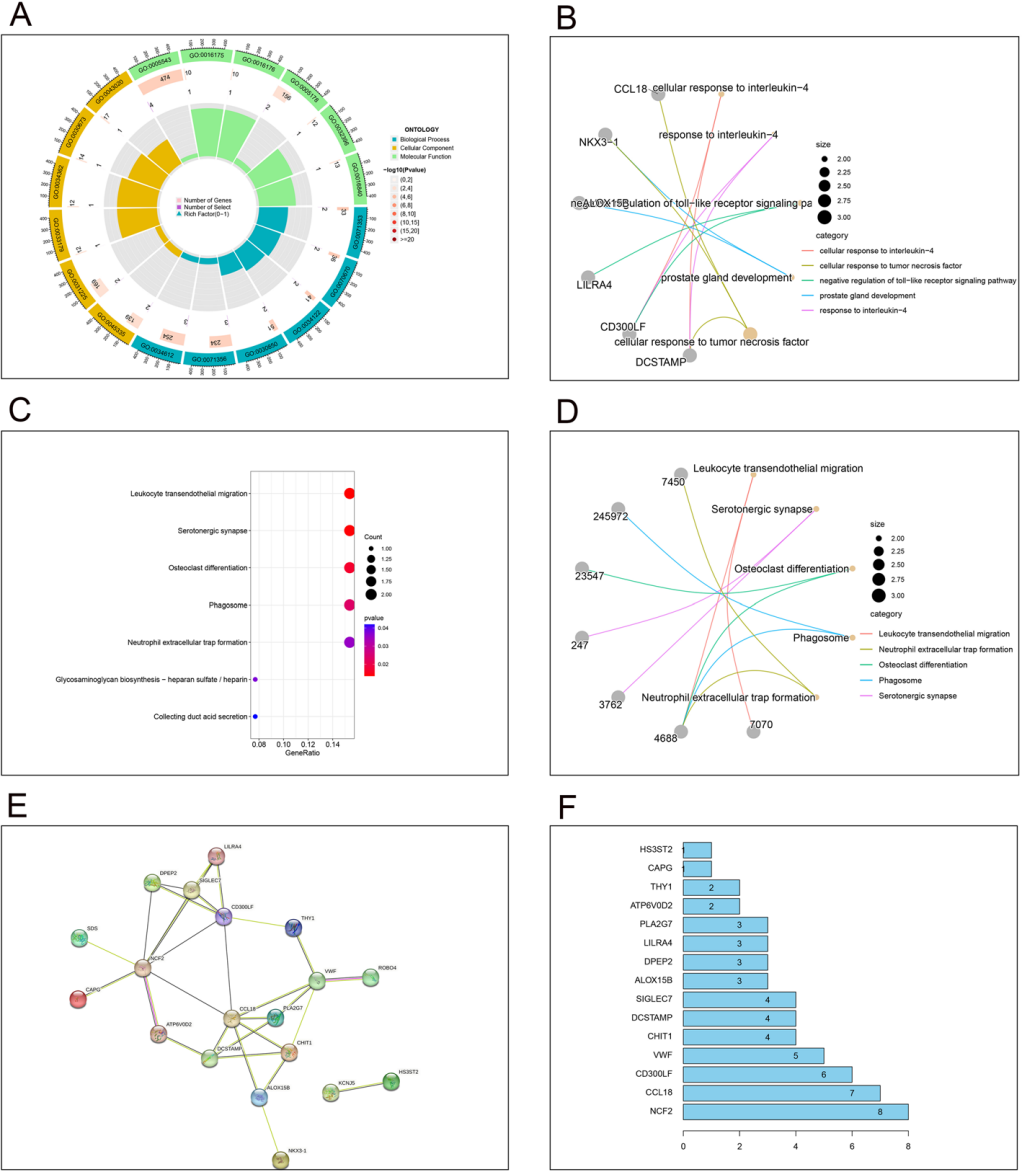
The result of functional enrichment analysis. ** A**, **B** GO function enrichment analysis results. From outside to inside, the first circle represents the ID of the GO, the second circle represents the number of genes on each GO, the color of the second circle represents the significance of the enrichment, the redder the color means the more significant the enrichment, the third circle represents the number of co-expressed genes, and the fourth circle represents the heat ratio of genes. **C**, **D** KEGG function enrichment analysis results. The color of the bar graph represents the P-value, the color change from light to dark means that the P-value becomes larger gradually, and the size of the endpoints represents the number of genes enriched in the pathway, the larger the endpoints the greater the number of enriched genes. **E **Protein protein interaction network, Protein interaction network results. The nodes represent genes, and the line between nodes indicates that two genes have protein interactions with each other. **F** Statistics of the number of protein interactions

We conducted GO and KEGG functional enrichment analysis on M2 macrophage-related genes to determine the pathways of these genes. By GO enrichment analysis, we found that M2 macrophage-related genes were mainly enriched in the following pathways (cellular response to interleukin-4; response to interleukin-4; cellular response to tumor necrosis factor; response to tumor necrosis factor; monocyte chemotaxis; regulation of pattern recognition receptor signaling pathway; inhibitory MHC class I receptor activity). These genes were then analyzed by KEGG enrichment analysis and mainly enriched in the following pathways (Leukocyte transendothelial migration; Serotonergic synapse; Osteoclast differentiation; Phagosome; Neutrophil extracellular trap formation; Glycosaminoglycan biosynthesis—heparan sulfate/heparin; Collecting duct acid secretion), and the results are shown in Fig. 3A–D. Furthermore, protein interaction network analysis was used to clarify potential relationships between these genes. After setting the filtering conditions and filtering to individual nodes, a protein interaction network map consisting of 19 nodes and 60 relationship pairs was obtained, and the results are shown in Fig. 3E, F.

### Construction of prognostic models

**Figs. 4:**
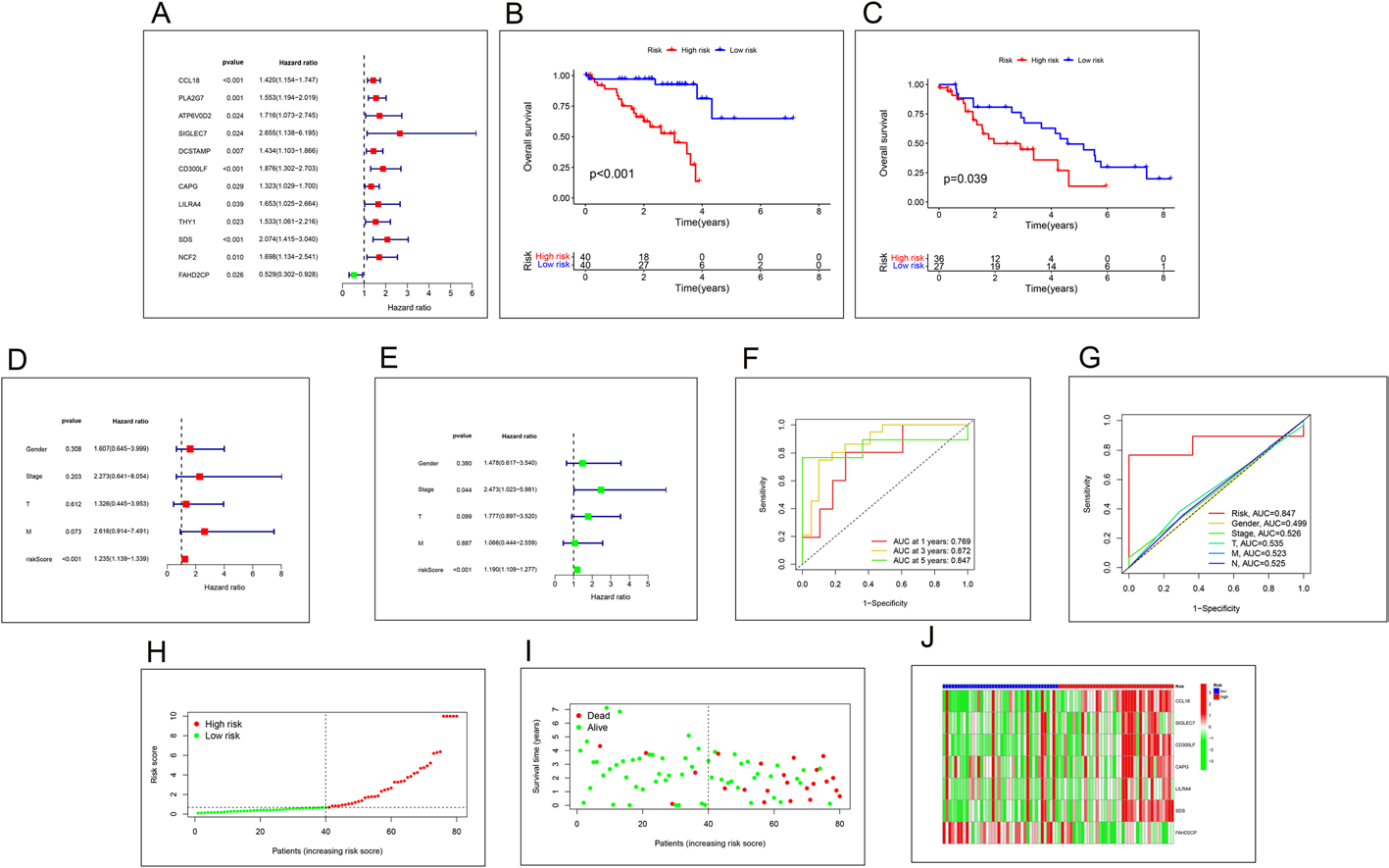
**A** Genes associated with prognosis obtained by univariate Cox regression analysis, Red means HR value is greater than 1, green means HR value is less than 1. **B**, **C** Survival curves. As survival time increased, the survival rate of the high-risk group was significantly lower than that of the low-risk group, B is the training set with data from the TCGA database and C is the training set with data from the GEO database. **D**, **E** The forest plots for single and multi-factor were significantly different only for risk values with p-values less than 0.05. **F**, **G** ROC curves. **F** ROC curve of survival time, ROC curve of clinical data. **H**, **I** Risk curves. The number of deaths is higher in high-risk patients than in low-risk. **J** The heatmap of risk scores. CCL18, SIGLEC7, CD300LF, CAPG, LILRA4, SDS are high-risk genes, FAHD2CP is low-risk gene

We screened for possible prognostic genes using one-way Cox regression on samples from the training cohort in the TCGA database. Genes that showed significance (p-value < 0.05) in Cox analysis were considered potential prognostic genes, and 12 prognosis-related genes were obtained by HR value and P-value screening. The results are displayed in Fig. 4A. Using multifactorial Cox regression analysis, we developed prediction models using seven target genes shared across samples in the TCGA and GEO databases. Moreover, risk values were obtained for each sample based on the prognostic models, dividing patients into low- and high-risk groups, using the median risk score as the cut-off point.

### Survival analysis

Combining each sample’s risk value and clinical data in our constructed model, we performed a survival analysis, and from the results, we can see that the survival time of patients in the training and validation groups is significantly different between the high and low-risk groups. Moreover, it can be observed that our constructed model has a good role in survival prognosis. The results are shown in Fig. 4B, C. To further verify the accuracy of the model prediction, we performed independent prognostic analysis by grouping the samples into different sexes and stages respectively, and the risk values of our prognostic model have good prediction results in both univariate and multifactorial prognostic analysis, and the p-values are less than 0.01. survival analysis, the results are shown in Fig. 4D. To validate the accuracy of model predictions further, we assessed the accuracy of our model across multiple groups by separating the samples and performing survival analysis individually for different sexes and different stages. The outcomes are depicted in Fig. 4E. We further demonstrated the prediction accuracy of the prediction model at survival times of 1-, 3-, and 5-years by using ROC and risk curves, where the area under the ROC curve was (AUC at 1 year: 0.769; AUC at 3 years: 0.872; AUC at 5 years: 0.847), respectively. The results are shown in Fig. 4F, G. The risk curve shows that the number of deaths is higher in high-risk patients than in low-risk. The results are shown in Fig. 4H–J.

### Survival analysis of clinical subgroups

**Figs. 5:**
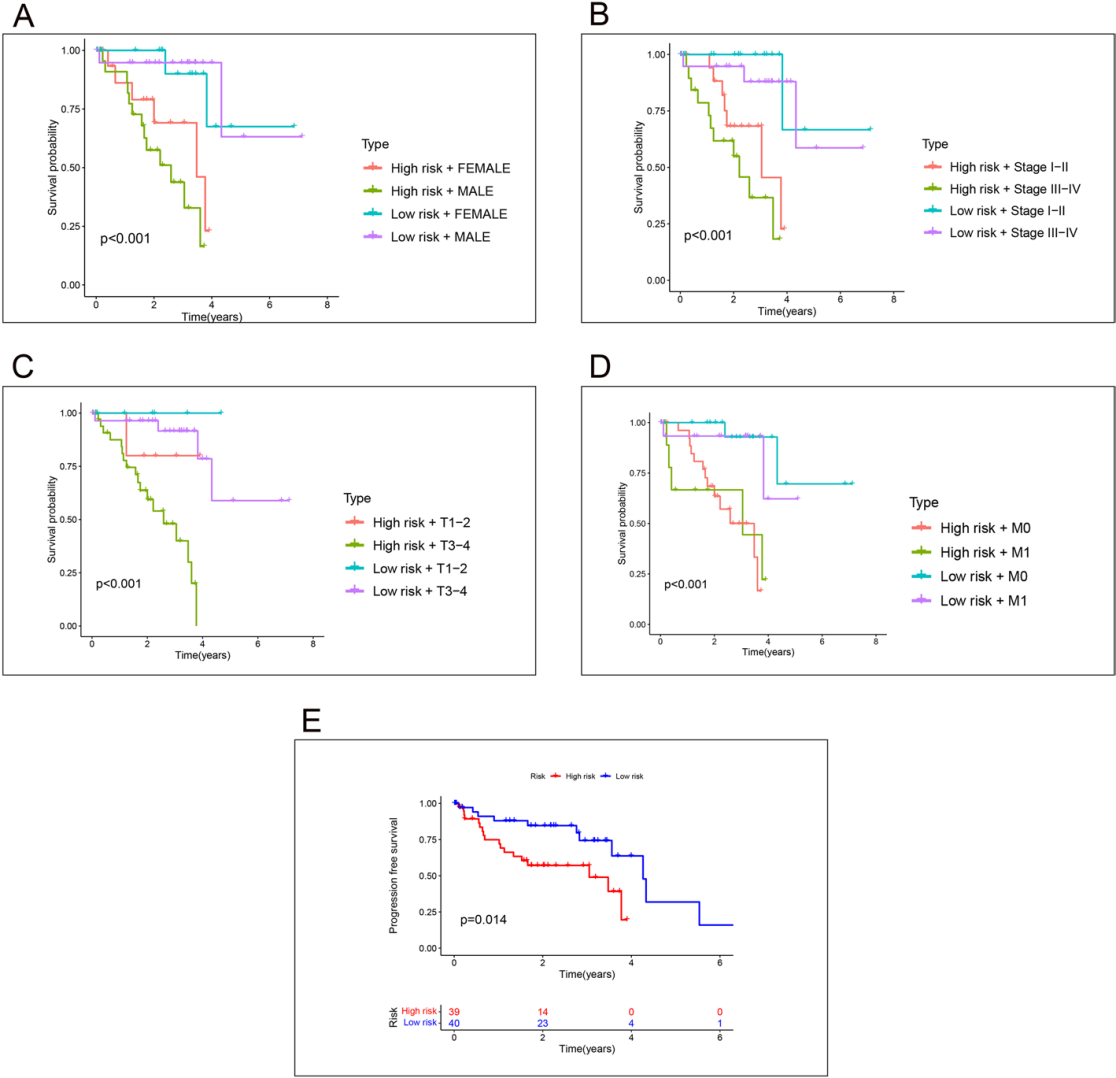
Survival analysis of clinical subgroups. ** A**–**D** Results of survival analysis between high and low risk groups by gender, stage, T-stage and M-stage. **E** Results of PFS analysis, Results of progression-free survival were significantly different between high and low risk groups

We grouped the sample’s clinical data and compared the prognostic model’s prediction results between the different subgroups by combining the sub permits of the clinical data. There are considerable disparities in survival between high- and low-risk subgroups of gender, grade, and T, M staging. The results are shown in Fig. 5A–D.

### PFS analysis

Combining the clinical data of pan-cancer in the TCGA database and the risk values of the samples obtained by our model construction, we further compared the survival differences between the high and low-risk groups in progression-free survival status by dividing them into high and low groups by the median of risk values. According to the results of our model grouping, there is a substantial difference in progression-free survival between the high and low-risk groups regarding survival time. The results are shown in Fig. 5E.

### GSEA functional enrichment analysis

**Figs. 6:**
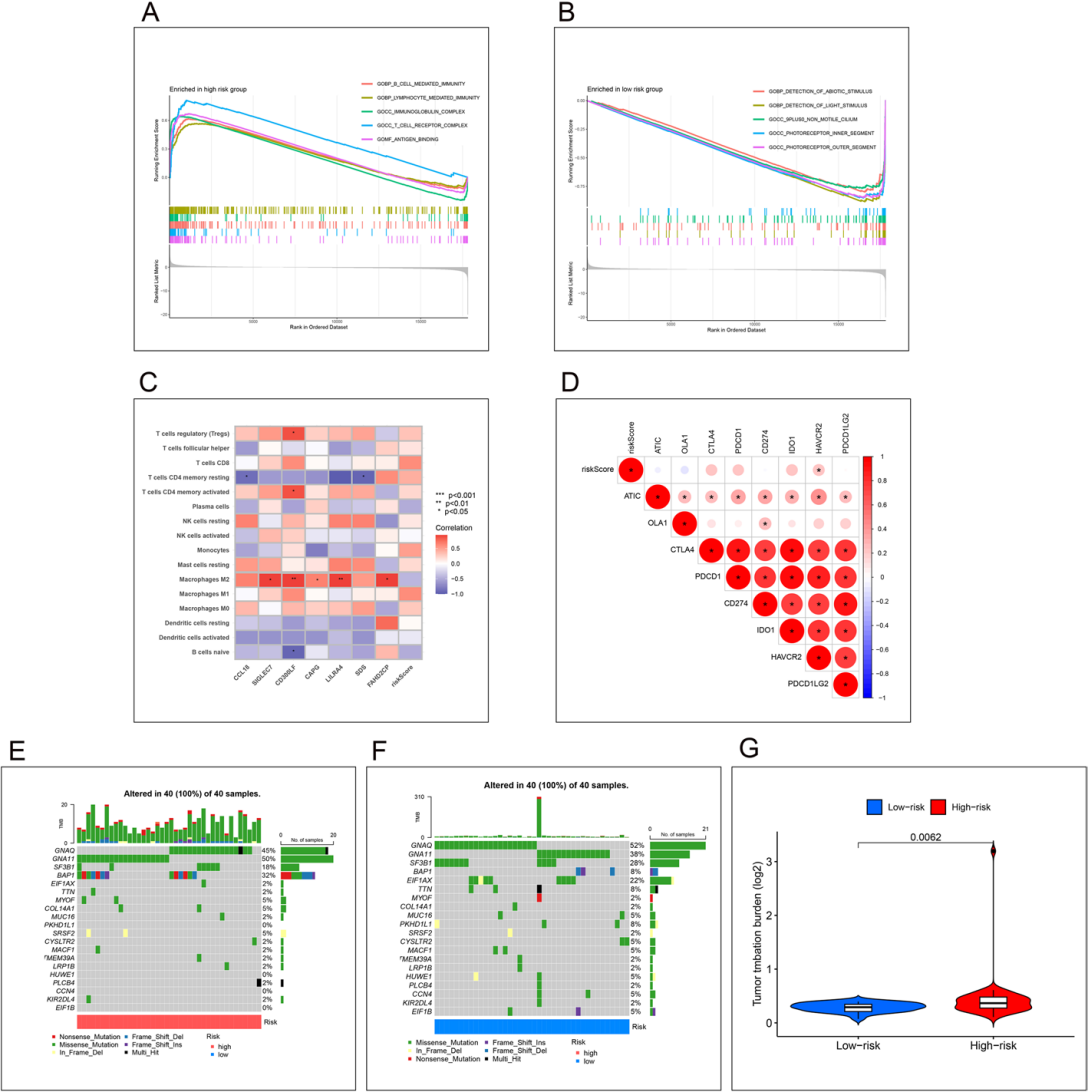
**A**, **B** Results of GSEA functional module analysis. The peak of the curve at the top indicates pathways that are clearly expressed in the high-risk group, and the peak of the curve at the bottom indicates pathways that are clearly expressed in the low-risk group. **C** Immune cell correlation analysis. The redder the color, the stronger the correlation. **D** Immune checkpoint correlation analysis. The expression of HAVCR2 had the strongest correlation with the value at risk. **E**–**G** Waterfall plot of tumor mutation load, the mutation frequency of the target genes we obtained is higher in the high risk group than in the low risk group

The GSEA functional enrichment analysis was performed by combining the risk value of each sample and the gene expression matrix in the samples. Moreover, the pathways that were significantly expressed in the high-risk group were as follows: (GOBP_B_CELL_MEDIATED_IMMUNITY; GOBP_LYMPHOCYTE_MEDIATED_IMMUNITY; GOCC_IMMUNOGLOBULIN_COMPLEX; GOCC_T_CELL_RECEPTOR_COMPLEX; GOMF_ANTIGEN_BINDING) and the pathways that were significantly expressed in the low-risk group were (GOBP_DETECTION_OF_ABIOTIC_STIMULUS; GOBP_DETECTION_OF_LIGHT_STIMULUS; GOCC_9PLUS0_NON_MOTILE_CILIUM; GOCC_PHOTORECEPTOR_INNER_SEGMENT; GOCC_PHOTORECEPTOR_OUTER_SEGMENT), and the results are shown in Fig. 6A, B.

### Immune cell correlation analysis

We obtained the strength of correlation with different target genes by co-expression analysis of immune cells with modeling target genes, respectively, and the results are shown in Fig. 6C.

#### Immune checkpoint correlation analysis

Co-expression analysis of the expression of immune checkpoint-related genes and the risk value of the sample yielded immune checkpoint genes correlated with the risk of the sample. It is possible to see the degree of the association between immune cells and the risk value derived from the model development, with HAVCR2 having the strongest correlation with the risk value. The results are shown in Fig. 6D.

### Analysis of differences in tumor mutation load

By using the tumor mutation load data of the samples in the TCGA database, combined with the risk values of the samples, we analyzed the differences in mutation load in the high and low-risk groups. Furthermore, we analyzed the mutation differences of the modeled genes between the high and low-risk groups, and from the results, it can be observed that the mutation frequency of the target genes is higher in the high-risk group than in the low-risk group. The results are shown in Fig. 6E–G.

#### Drug sensitivity analysis

**Fig. 7:**
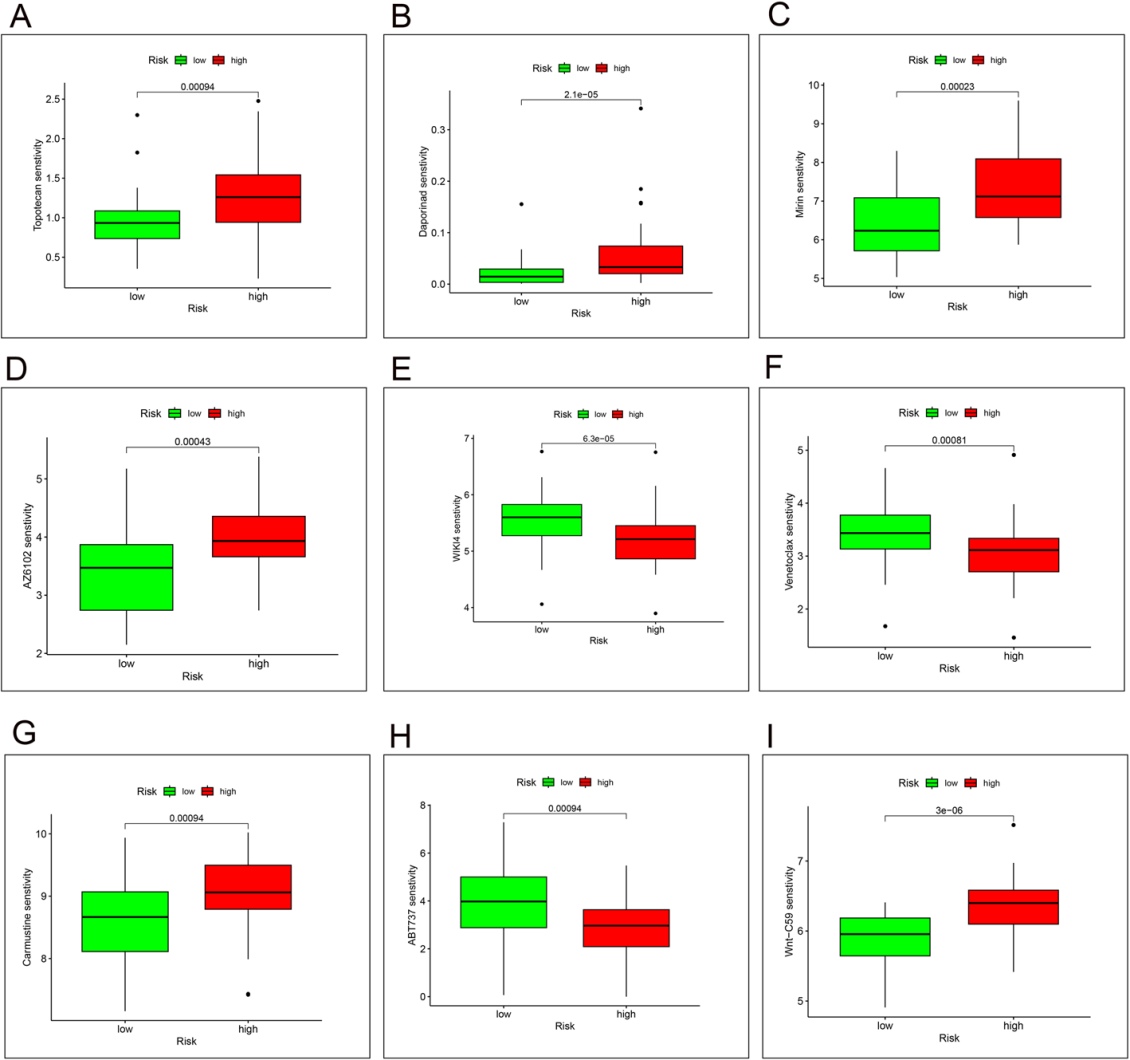
The results of drug sensitivity analysis. **A**–**I** Results of differences in drug sensitivity of 9 drugs in high and low risk groups, where Topotecan, Daporinad, Mirin, AZ6102, Carmustine and Wnt-C59 have a greater sensitivity score in the high-risk group than in the low-risk group, and WIKI4, Venetoclax, ABT737 have a smaller sensitivity score in the high-risk group than in the low-risk group

We scored each sample’s drug sensitivity in conjunction with the database’s data file on drug sensitivity, and then, in conjunction with each sample’s risk value, we analyzed the sensitivity of high-risk and low-risk groups to various drugs. By screening the results and removing any without differences, we were left with the data for nine drugs with different drug sensitivity results in high- and low-risk groups. The results are shown in Fig. 7A–I.

## Discussion

Uveal melanoma is a malignant tumor in the uvea’s melanocytes. It is the most common, and despite new treatments, the prognosis remains poor, with up to 50% of patients developing metastases without effective treatment options [15]. Unlike cutaneous melanoma, uveal melanoma is considered an “immune escape” tumor [16], because of its low mutation burden and unique immunosuppressive microenvironment [17]. Advanced cancers still have few therapy options available today. Immunotherapies provide hope for effectively managing many advanced diseases, but their therapeutic efficacy is suboptimal and greatly varies across individuals. Tumor-associated macrophages (TAMs) are a major component of the tumor microenvironment (TME) [6], this condition is usually associated with poor prognosis and treatment resistance (including immunotherapy) [18, 19], Therefore, a deeper comprehension of the intricate function of tumor macrophages in immunotherapy control may offer fresh perspectives on TME and lead to additional research into immunotherapy for advanced tumors. This study explored intra-tumor immune infiltration in uveal melanoma using The Cancer Genome Atlas (TCGA) and Gene Expression Omnibus (GEO) databases and the CIBERSORT algorithm [17, 18].

With the recent progress and wide application of second-generation sequencing (NGS) technology, more transcriptomic data of tumor tissues are rapidly accumulated. TCGA and GEO databases are the most applied public databases containing transcriptional data of various tumor tissues [20]. This makes it possible to analyze the outcomes of immune cell infiltration in tumor tissues using a significant amount of transcriptional data and additional comparative analyses. This substantially simplifies the technique of relying exclusively on tissue section staining, whether by transcriptome sequencing findings or by further obtaining the results of immune cell infiltration analysis in target tumor tissues from single-cell RNA sequencing (scRNA-seq) data in different cancer settings. There are currently recognized methods for calculating immune cell infiltration in the following collections, including CIBERSORT [21], ESTIMATE [22], quanTIseq [23], TIMER [24], IPS [25], MCPCounter [26], xCell [27] and EPIC [28]. CIBERSORT is the most recognized method for detecting 22 immune cells in TME, which can analyze cell biomarkers and therapeutic targets in RNA mixtures on a large scale with high accuracy. In addition to obtaining the relationship between immune cell infiltration and patient prognosis from the macro level, different types of immune cell infiltration can also be thoroughly understood about tumor growth and invasion from the micro level by using the CIBERSORT calculation method to obtain the results of immune cell infiltration in tumor tissue and then conducting joint analysis on clinical data of patients in the TCGA database. To provide research directions for finding better immune targets and therapeutic drugs. Applying the CIBERSORT algorithm to analyze the immune infiltration of tumor tissues in the TCGA database, the infiltration scores of various immune cells in renal clear cell carcinoma were obtained, and the immune cell types related to prognosis were obtained. Moreover, the infiltration scores of immune cells in other tissues, such as head and neck squamous cell carcinoma, gastric cancer, and other tumors [29–31]. Particularly the infiltration of M2 macrophages was studied about tumor growth and invasion; this provides a reference for our research [32–34].

We evaluated the uveal melanoma patients’ prognosis by M2 macrophage immune cell-related genes combined with tumor patients’ clinical data. We built a prognostic model by M2 macrophage signature genes, with CCL18, SIGLEC7, CD300LF, CAPG, LILRA4, SDS, and FAHD2CP as the final modeled genes as our study focus. CCL18 (C-C Motif Chemokine Ligand 18), Tumor-associated macrophages (TAMs) are vital in the malignant tumors’ development. Studies have shown that TAMs promote uveal melanoma metastasis by secreting CCL18 ^35^. This chemokine attracts naive T lymphocytes to activated macrophages in dendritic cells and lymph nodes. It may play a role in humoral and cell-mediated immune responses [36]. In the presence of chemokines, the cellular interference mechanism between pericytes and uveal melanoma is more prominent, resulting in the conversion of pericytes into activated fibroblasts and laying the groundwork for the metastatic spread of tumor cells and fibrosis development [37]. SIGLEC7 (Sialic Acid Binding Ig Like Lectin 7), whose main relevant pathways include the natural immune system and class I MHC-mediated antigen processing and presentation [38]. Recent use of Siglec-7 as a therapeutic target for glyco-immune checkpoint and T-cell driven diseases and cancers [39]. CD300LF (CD300 Molecule Like Family Member F), the gene encodes a member of the CD300 protein family. Members of this family are cell surface glycoproteins with a single IgV-like extracellular structural domain that regulates immune response and leukocyte functions like activation, proliferation, differentiation, migration, and immune function. They are considered potential targets for studying the development and progression of inflammation, infection, and other diseases [40]. CAPG (Capping Actin Protein and Gelsolin Like) This gene encodes a member of the actin regulatory protein gelatin/vimentin family. The encoded protein reversibly blocks the ends of actin filaments in a Ca^2+^ and phosphatidylinositol-regulated manner [41]. Cells and tissues from diffuse large B-cell lymphoma expressed CAPG at high levels. CAPG enhances the proliferation and invasion of diffuse large B-cell lymphoma cells, inhibits apoptosis, and activates the PI3K/AKT signaling pathway [42]. LILRA4 (Leukocyte Immunoglobulin Like Receptor A4) is a gene encoding an immunoglobulin-like cell surface protein, is expressed primarily on plasmacytoid dendritic cells (PDCs), and regulates these cells’ function in the immune response [43, 44]. SDS (Serine Dehydratase), This gene encodes one of three enzymes involved in the metabolism of serine and glycine. l-Serine dehydratase converts L-serine to pyruvate and ammonia and requires pyridoxal phosphate as a cofactor [45]. FAHD2CP (Fumarylacetoacetate Hydrolase Domain Containing 2 C), widespread expression in human testis and brain tissues. Expressed also in gastric cancer tissues in transcriptome sequencing [46]. The functional enrichment analysis shows that, based on GO enrichment analysis, we found that M2 macrophage-related genes are mainly enriched in the following pathways (cellular response to interleukin-4; cellular response to tumor necrosis factor; response to tumor necrosis factors; monocyte chemotaxis; regulation of pattern recognition receptor signaling pathways; inhibitory MHC class I receptor activity), and by KEGG enrichment analysis, we found that these genes were mainly enriched in the following pathways (Leukocyte transendothelial migration; Serotonergic synapse; Osteoclast differentiation; Phagosome; Neutrophil extracellular trap formation; Glycosaminoglycan biosynthesis—heparan sulfate/heparin; Collecting duct acid secretion). The enrichment results of these genes focus on the aggregation of monocyte macrophages and pathways like polarization, and interleukin 4 is the cytokine most typically used to induce monocyte-macrophage polarization to M2-type macrophages in vitro [47, 48]. For monocyte chemotaxis 49–51; the regulatory pathway of pattern recognition receptor signaling pathway also indicates the chemotaxis generated by tumor cells in the body, recruiting monocyte macrophages in the immune system to further play a role in tumorigenesis development. Prior research has shown that the number and complexity of tumor trophoblast vessels can predict tumor growth rate and invasive metastasis, determining the tumor patient’s prognosis [52, 53], M2-type macrophages have a tumor-promoting angiogenic function, and tumor-associated macrophages and their cytokines appear responsible for increased tumor aggressiveness [54, 55]. The tumor-favorable and angiogenesis-promoting effects are directly attributable to the M2-dominated tumor microenvironment, suggesting a plausible mechanism for the tumor-promoting actions of M2-type macrophages. We established a prognostic model by these macrophage-associated target genes and tested the model’s reliability by survival analysis. Through multiple functional analyses, the potential functions of these genes in the development of uveal melanoma were identified, setting the groundwork for future research.

## Conclusions

In this study, we explored the intra-tumor immune infiltration in uveal melanoma by the CIBERSORT algorithm by analyzing the sequencing results of uveal melanoma from TCGA and GEO public databases. We assessed the prognosis of uveal melanoma patients by the M2 macrophage immune cell infiltration (ICI) score, built a prognostic model by the characteristic genes of M2 macrophages, investigated the pathways of action of these characteristic macrophage genes by functional analysis, and validated our prediction by combining tumor mutational load, immune checkpoint, and drug sensitivity, respectively. Combining tumor mutational load, immunological checkpoint, and drug sensitivity confirmed the validity of our model, which serves as a benchmark for future research on uveal melanoma and immunotherapy.

## Supplementary Information

**Additional file 1**. Supplementary Figures legends.

**Additional file 2**. Analysis of M1 type macrophage related gene expression levels.

**Additional file 3**. The result of single-cell analysis.

## Data Availability

The original data comes from TCGA (https://portal.gdc.cancer.gov/) and GEO (https://www.ncbi.nlm.nih.gov/geo/query/acc.cgi?acc=GSE22138) public database, and the data is accurate.

## Abbreviations

TCGA: The Cancer Genome Atlas
GEO: Gene expression omnibus database
GO: Gene Ontology analysis
KEGG: Kyoto Encyclopedia of Genes and Genomes (KEGG) pathway analysis
GSEA: Gene Set Enrichment Analysis
