# Supplementary material for: Prognostic analysis of uveal melanoma based on the characteristic genes of M2-type macrophages in the tumor microenvironment
Source: BMC Bioinformatics. 2023 Jul 11;24:280. doi: 10.1186/s12859-023-05396-9 (PMC10334534; doi:10.1186/s12859-023-05396-9)
Supplement: Supplementary file 1 — Additional file 1. Supplementary Figures legends. [file 12859_2023_5396_MOESM1_ESM.docx]

Supplementary Figure 1

**Analysis of M1 type macrophage related gene expression levels**

By using the same co expression analysis method, we further analyzed the infiltration results of M1 type macrophages in tumor tissue and obtained M1 type macrophage related genes. After setting filtration conditions and P-values, we obtained M1 type macrophage related genes. Combined with the risk values of the previously obtained samples, we compared and analyzed M1 type macrophage related genes in the high and low risk groups.

Supplementary Figure 2

**Single-cell analysis**

Analysis of specific genes at single-cell level of uveal melanoma was conducted using the Tumor Immune Single-cell Hub (TISCH) project. Then, we identified two uveal melanoma single-cell cohorts (GSE139829 and GSE138433) to perform the single-cell analysis of CCL18, SIGLEC7, CD300LF, CAPG, LILRA4, SDS, and FAHD2CP. However, the FADH2CP was not found in the gene symbol list of GSE139829 and GSE138433. For other genes, results of GSE139829 and GSE138433 indicated that CAPG, CCL18, CD300LF, LILRA4, SDS and SIGLEC7 were mainly expressed in the monocyte/macrophages.
