# Supplementary figures and images for: Prognostic analysis of uveal melanoma based on the characteristic genes of M2-type macrophages in the tumor microenvironment
Source: BMC Bioinformatics. 2023 Jul 11;24:280. doi: 10.1186/s12859-023-05396-9 (PMC10334534; doi:10.1186/s12859-023-05396-9)

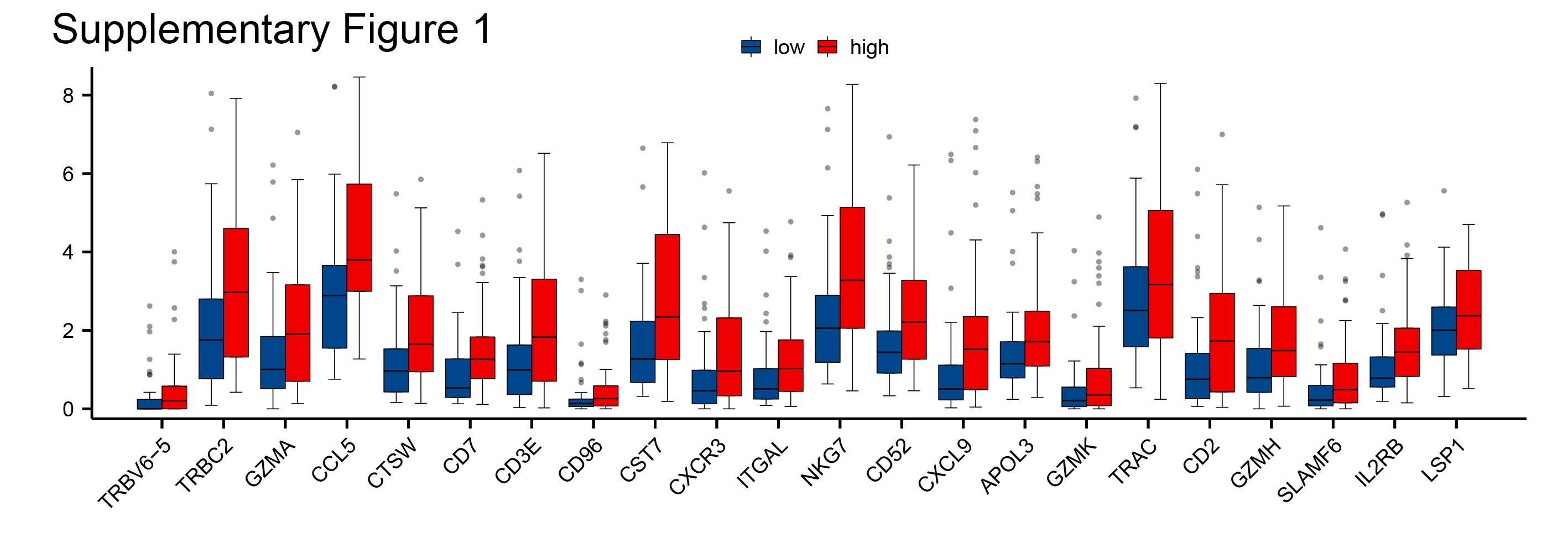

Supplement: Supplementary file 2 — Additional file 2. Analysis of M1 type macrophage related gene expression levels. [file 12859_2023_5396_MOESM2_ESM.jpg]
